# Potentially Preventable Hospitalisation Related to Severe Third Molar Disease: A Systematic Review

**DOI:** 10.3390/dj14080503

**Published:** 2026-08-08

**Authors:** Terence Lau, Albert Yaacoub, Stephen Cox, Mafaz Ullah

**Affiliations:** 1 Discipline of Oral Surgery, Sydney Dental School, Faculty of Medicine and Health, University of Sydney, Sydney, NSW 2750, Australiaalbert.yaacoub@health.nsw.gov.au (A.Y.); stephen.cox@sydney.edu.au (S.C.); 2 Nepean Centre for Oral Health, Nepean Blue Mountains Local Health District, Kingswood, NSW 2747, Australia; 3 School of Nursing and Midwifery, Western Sydney University, Locked Bag 1797, Penrith, NSW 2751, Australia

**Keywords:** wisdom teeth, third molar, dental infection, pericoronitis, postoperative infection, hospitalisation

## Abstract

**Background/Objectives:** One of the most common oral surgery procedures performed in dentistry is the removal of third molar teeth (3M). Complications of both retention and removal of third molars are associated with potentially preventable hospitalisations (PPH). This study aims to systematically review the published literature to identify demographics, aetiology, incidence, associated factors, length of hospital stay (LOS) and variables influencing the outcomes of PPH arising from 3M. **Methods:** An electronic literature search was performed using Medline and Embase via Ovid in May 2024, and relevant studies were analysed for demographics, aetiology, incidence, related factors and LOS associated with PPH resulting from 3M. **Results:** Nineteen publications with sample sizes ranging from 37 to 3549 patients per study were identified. Two studies reported 101 patients described PPH specifically related to 3M. The remaining 17 studies described 6153 oral health-related PPH, predominantly arising from broader odontogenic infection cohorts, of which 1654 (26.9%) PPH were associated with 3M. The mandibular 3M was implicated in 98.5% of 3M-related PPH. Pericoronitis and postoperative infections following 3M removal were reported as the leading causes of hospitalisation, whereas data on dental caries were not reported. Similarly, most studies did not report detailed information on demographics, aetiology, or outcomes specific to third molars. **Conclusions:** Available indirect data shows that 3M-related dental infections, including post operative infections, contribute to oral health-related PPH. There are significant evidence gaps in third molar-specific PPH data. Further studies with standardised reporting focusing on third molar-related hospitalisations are required to identify factors influencing clinical outcomes and to inform evidence-based clinical decision-making regarding the removal or retention of third molars.

## 1. Introduction

Wisdom teeth, or third molar teeth (3M), are the last set of molars erupting into the dental arch, typically in the late teenage years or early twenties [1] The 3M is estimated to be present in over 96% of humans, and is associated with conditions such as pericoronitis, dental caries, periodontitis, cysts, and tumours of the jaws [2,3]. In adults aged 30–93 years, 44% individuals were found to retain at least one 3M, with 84% of 3M classified as diseased [4].

Dental infections, also known as odontogenic infections, commonly arise from bacterial invasion into the pulpal tissues from dental caries or inflammation of the tooth-supporting structures associated with periodontal diseases. Dental infection arising from maxillary 3M may involve the buccal space, maxillary sinus, orbit, infratemporal fossa and rarely cavernous sinus [3,5,6]. In contrast, dental infections originating from the mandibular third molar (M3M) may spread to buccal, sublingual, submandibular, retro- pharyngeal, sub-masseteric and pterygomandibular spaces, potentially resulting in airway compromise, sepsis, necrotizing mediastinitis or death [3,5,7]. Similar infections may arise following the removal of 3M as postoperative dental infections. Spreading infection arising from dental caries, periodontal dental diseases, or postoperative complications requires management in a hospital setting [3,5,8].

Dental infections requiring hospitalisation are considered PPH, as the major contributors, dental caries, pericoronitis and periodontitis, are preventable with early disease management in a primary dental care setting [5,9]. PPH are associated with both patient morbidity and costs for the healthcare system, with 87,410 reported dental PPH in 2022–2023 in Australia [10]. The financial burden of odontogenic infections is significant. According to a study from the United States, the average hospital cost for managing an acute dental infection was 28,841 USD per patient [11]. An Australian study estimated an average cost of $12,228 Australian dollars per patient, with $4500 Australian dollars per day, from 2006 to 2014 for the management of odontogenic infections [12]. Data from National Health Service hospitals in England revealed an 8-fold increase in patients admitted to hospital for drainage of dental abscesses in 2019–2020 compared to 2000 [13].

Management of 3M continues to be an area of controversy internationally. In Australia, there are no prescribed treatment protocols for the management of 3M, with dentists and specialists encouraged to balance the surgical risks with potential benefits before deciding, in consultation with patient preferences, whether surgical intervention is indicated [14]. Guidelines published by the National Institute for Health and Care Excellence (NICE) in the United Kingdom in 2000, restricted the surgical removal of 3M to unrestorable caries, non-treatable pulpal or periapical pathology, cellulitis, abscesses and osteomyelitis, resorption, cysts, and tumours [15]. Following an initial drop of 30% in the number of 3M removals after introduction of the NICE guidelines, subsequent years saw a 97% increase in 3M extraction, with the mean age of patients undergoing 3M removal increasing from 25.5 years to 31.9 years, and more associated with pathology secondary to dental caries [16,17]. More recently published parameters of care by the Royal College of Surgeons of England, Faculty of Dental Surgery, have suggested that this may not be in the patient’s best interests, merely delaying the inevitable surgery [18]. Previous studies have also reported the age of 25 to be the critical age after which there is an increase in post-operative complications, including surgical site infection (early/late onset), periodontal complications, permanent nerve injury and jaw fracture, following 3M extraction [19].

Conversely, the American Association of Oral and Maxillofacial Surgeons recommends surgical management of 3M associated with disease or considered to be at high risk of developing disease [20]. Adding to the uncertainty, a recent Cochrane review found insufficient evidence from randomized controlled trials for or against the prophylactic removal of 3M, encouraging clinicians to consider patient values and clinical expertise in third molar decision-making [21].

Dental infections arising from 3M, and resulting in PPH have been reported [3]. A systematic review would enable a more comprehensive understanding of the available data. The aim of this review is to answer the question, “What is the incidence, demographics, aetiology, associated factors, LOS and variables influencing the outcomes of potentially preventable hospitalisations arising from third molar teeth in adults?” By systematically reviewing the published literature, this study seeks to identify evidence gaps that may inform future preventive strategies and guide clinical decision-making regarding the retention or removal of third molars.

## 2. Materials and Methods

### 2.1. Study Registration

The systematic review was prospectively registered on PROSPERO (CRD42024549405), with study methods established a priori.

### 2.2. Search

An electronic search of the literature was conducted using the MEDLINE(R) ALL and Embase Classic databases via Ovid. Search terms included both MeSH and Emtree subheadings described below, in addition to keyword searching. Furthermore, the reference lists of relevant articles were screened to identify additional eligible publications. The final search was completed on 24 May 2024.

### 2.3. MeSH Headings

“Molar, Third/or Tooth, Impacted/and Dental caries/or Periapical abscess/or Periapical periodontitis/or Periodontitis/or Pericoronitis/or Focal Infection, Dental/or Abscess/or Bacterial Infections/or Tooth Diseases/or Infections/or Cellulitis/or Abscess/or Osteomyelitis/or Ludwig’s angina/or Retropharyngeal abscess/or Periodontal abscess/or Surgical wound infection/or Postoperative complications/or Cavernous sinus thrombosis/or Wounds and injuries/or Dentigerous cyst/or Odontogenic cysts/or Odontogenic tumors/or Ameloblastoma/or Jaw neoplasms/and Hospitalization/or Inpatients/”.

### 2.4. Emtree Headings

“Third molar/or Mandibular third molar/and Dental caries/or Chronic periodontitis/or Periodontitis/or Gingiva disease/or Tooth periapical disease/or Periapical periodontitis/or Tooth infection/or Bacterial infection/or Infection/or Abscess/or Mouth infection/or Surgical infection/or Cellulitis/or Orbital cellulitis/or Ludwig angina/or Head and neck infection/or Retropharyngeal abscess/or Mediastinitis/or Lemierre syndrome/or Periapical abscess/or Orbit infection/or Postoperative infection/or Cavernous sinus thrombosis/or Sepsis/or Osteomyelitis/or Dentigerous cyst/or Jaw disease/or Odontogenic cyst/or Keratocyst/or Ameloblastoma/or and Hospitalization/or Hospital admission/or Hospital patient/.”

### 2.5. Study Selection

All records were collated in a single EndNote library and duplicates removed. Publications were screened according to inclusion and exclusion criteria by two independent reviewers (TL and MU), and disagreements between reviewers were planned to be resolved by discussion with a third reviewer (SC). Inter-reviewer agreement during study selection was assessed using Cohen’s kappa. There were no disagreements between authors.

Supplementary Table S1 details studies excluded at the eligibility stage describing the principal reasons for exclusion.

Inclusion criteria comprised original hospital-based studies published in English, with published data on hospitalisation related to 3M in adult patients. Exclusion criteria included studies without 3M data, case reports and literature reviews. Most studies excluded at the “assessed for eligibility” stage considered hospitalisations from dental infections more generally, but did not publish data relating to 3M. Non-English studies were excluded due to difficulty in article translation and interpretation by reviewers, and the lack of availability and cost of professional translators.

### 2.6. Data Extraction

Relevant data were extracted by the first author (TL) and collated in Excel Microsoft 365 Apps for Enterprise (Microsoft Corporation, Washington, DC, USA). Data extraction was not performed in duplicate.

### 2.7. Quality Appraisal

Quality appraisal of the selected studies was conducted using the Joanna Briggs Institute (JBI) critical appraisal tool for case series, which comprises 10 questions addressing internal validity and risk of bias [22]. Case series may provide the best available evidence in cases where higher-quality study designs are unavailable, but they are more vulnerable to incomplete reporting. This tool is in active use by the JBI collaboration and has been reviewed by the JBI Scientific Committee, demonstrating face validity [23].

## 3. Results

**Figure 1:**
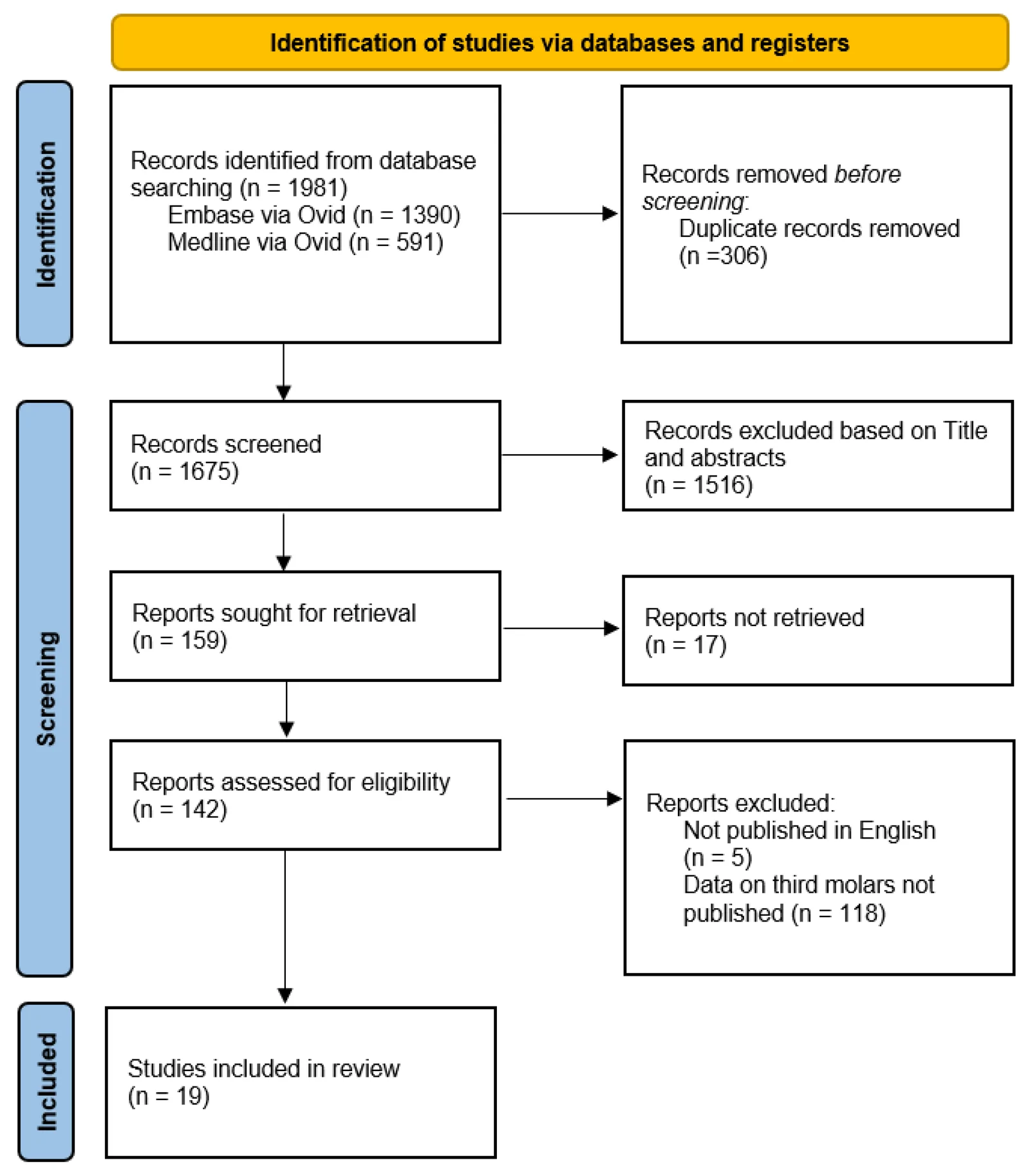
PRISMA [22] flow chart of literature search.

Nineteen studies were ultimately selected based on inclusion and exclusion criteria (Figure 1).

### 3.1. Characteristics of Studies

The 19 included studies (Table 1) were retrospective hospital-based audits published between 2006 and 2024, originating from a range of countries, including Australia, USA, Europe, India, and China, as well as Uganda and Brazil.

### 3.2. Hospitalisations Attributed to 3M

The included studies described 6593 dental infection-related hospitalisations.

The other 17 studies included 6153 PPH arising from dental infection more broadly [24,25,26,27,28,29,30,31,32,33,34,35,36,37,38,39,41]. Of these, 1654 (26.9%) PPH were attributed to 3M [24,25,26,27,28,29,30,31,32,33,34,35,36,37,38,39,41]. Third molar involvement ranged from 9.8% to 56.2% [24,28]. A total of 25 (1.5%) of the reported 3M-related PPH were described as arising from maxillary 3M (Table 2) [26,36,37,38,41].

### 3.3. Demographics

The included studies reported mean age ranges from 29.5 to 47.5 [33,34], with sample sizes of 37 to 3549 (Table 1) [27,41].

Kunkel et al. reported a gender split of 48 females to 52 males in their sample of PPH arising from 3M, with a mean age of 36.3 years [40]. The mean age of patients hospitalised for severe infection was 42 [40]. Patients hospitalised for jaw fracture following 3M extraction had a mean age of 46.1 [40].

The other studies reported only demographics of patients hospitalised from dental infection in general, but did not publish specific demographic data on 3M.

### 3.4. Aetiology

A majority of studies did not report the aetiology of 3M-related PPH and only identified the number of PPH related to 3M (Table 3).

#### 3.4.1. Pericoronitis

Five studies, with a total of 1351 cases, reported pericoronitis of the M3M as the cause of PPH in 220 (16.3%) of cases, with individual studies ranging from 5.9% to 26.7% [24,25,28,39,41]. Kunkel et al. reported pericoronitis of the M3M in 29% of 3M-related admissions [40].

#### 3.4.2. Postoperative Infection

Three studies with a total of 251 dental-related PPH [29,36,41] reported that 18 (7.2%) of these admissions occurred following postoperative infection after 3M extraction. Kunkel et al. reported postoperative infection to account for 51 (51%) of 3M-related admissions [40].

#### 3.4.3. Dental Caries

No studies explicitly described dental caries affecting the 3M as the cause of PPH.

#### 3.4.4. Other

In the only two studies describing 3M-related complications resulting in PPH, there were overlapping datasets of 100 and 55 patients, respectively, with 101 individual unique cases. Eleven cases of mandibular fracture, 5 cases of tooth displacement requiring retrieval, 3 cases of nerve injury, 1 case of osteomyelitis, and 1 case of postoperative haemorrhage were reported (Table 4).

### 3.5. Mean LOS

The mean LOS in the included studies (Table 3) considering dental infection overall ranged from 2.4 to 8.5 days [26,30]. 3M-related PPH were reported in only a very small subset of studies. Kunkel et al. reported mean LOS of 6.7 days following prophylactic 3M extraction, 7.9 days following therapeutic 3M extraction, and 7.1 days if the 3M was present at admission [40].

ICU Admission:

Three studies reported on ICU admission related to dental infection, with a total of 67 cases [29,31,35]. One study reporting on 52 ICU admissions found that 35 (67%) were related to M3M [31].

### 3.6. Outcomes

Five studies reported 40 cases of Ludwig’s angina (LA) as a complication of dental infection leading to PPH [24,26,30,37,38]. Saito and colleagues found that the M3M was involved in 60% (3/5) cases of LA [38], and the causative tooth was not described in the other four studies [24,26,30,37].

### 3.7. Mortality

The included retrospective studies reported nine deaths in total; 8 of these cases did not identify the tooth or cause of death [26,34,38]. Kunkel identified the cause of death as myocardial infarction due to secondary stent thrombosis on a background of coronary artery disease in a 77-year-old, 23 days after M3M removal [40].

## 4. Discussion

To the authors’ knowledge, this is the first systematic review of the published literature analysing the 3M specifically as a cause of PPH. It is important to note that only two studies provided third molar-specific hospitalisation data, with most included studies reporting broader odontogenic infection cohorts with partial attribution to third molars, limiting the reliability of any combined estimate. Quantitative synthesis or meta-analysis was not undertaken because the available evidence was limited and heterogenous. The included studies differed significantly in outcome reporting, with the aetiology of hospitalisation and third molar-specific hospitalisation data not being reported in most papers. This made statistical pooling inappropriate and potentially misleading. There was consensus in the included studies that the majority of 3M-related PPH arose from the M3M as opposed to the maxillary 3M, which is consistent with the anatomical proximity of the M3M to potential fascial spaces and the airway.

The studies by Rastenienne et al. and Rastenienne et reported overlapping datasets (285 patients) [26,37], as did the studies by Kunkel et al. and Kunkel et al. (54 patients) [3,40], leading to an overall total of 6254 dental-related hospitalisations. Cases from overlapping data were excluded to avoid “double counting” in aggregate data, but the outcomes from individual studies were still described individually in the result tables. Two studies reported 101 cases specifically related to 3M resulting in hospitalisations [3,40].

The heterogenous nature of different studies means that the definition of PPH may not be applied consistently throughout the individual studies. In the included studies, PPH may include a combination of odontogenic infection-related admissions, third molar-related admissions or admissions arising from postoperative complications. The reported proportion of 3M-related PPH (26.9%) should therefore be interpreted as a descriptive pattern of hospital admissions where a third molar was implicated, rather than an estimate of preventability. While many included studies clearly reported transparent demographics, inclusion criteria, and outcome reporting when assessed against the JBI critical appraisal tool, incomplete third molar-specific data and inconsistent outcome reporting still limit the overall quality of evidence.

In one study, the mean age of patients hospitalised following prophylactic removal (27) was younger compared to the mean age of patients hospitalised for severe dental infection (42) and jaw fractures (46) [40]. This was consistent with previous reports describing an increase in complications following 3M removal after the age of 25 [21].

Those studies reporting on 3M-related PPH only identified the numbers of 3M implicated in dental infection, but did not clarify the aetiology, so it is unclear if these cases arose due to dental caries, periodontal disease, pericoronitis, postoperative infection, or infected cysts or tumours of the jaws. With data from the United Kingdom suggesting that 3M removal is shifting to older adult population groups, and more likely to be associated with caries [16], it was interesting that none of the included studies presented explicit data for untreated dental caries of the 3M as a cause for dental infection leading to PPH. This is surprising, as pulpal disease was the most common dental cause for maxillofacial space infections overall, accounting for between 64% to 80.6% of total hospitalisations [24,25,37,38]. Only two studies with a shared dataset described the aetiology of each of the included 3M cases resulting in hospitalisation [3,40], identifying postoperative infection as a cause of hospitalisation at a much higher rate than all other included studies. Boffano et al. and Flynn et al. did not describe whether cases of hospitalisation for postoperative dental infection arose from therapeutic extraction with pre-existing dental infection, or following prophylactic extraction of teeth [36,41]. 

In 102 patients hospitalised with dental infection, Katoumas et al. found that all patients admitted with postoperative infection underwent therapeutic extraction with pre-existing pericoronitis and reported no hospitalisations following prophylactic 3M removal [27]. This corroborates with the results of Kunkel et al., who reported 46% of complications of 3M extraction resulting in hospitalisation to arise following therapeutic extraction [3]. In the entire study group, there were only 15 published incidences of hospitalisation following prophylactic 3M extraction [3], suggesting that this is not a common cause of PPH.

A further important limitation is the lack of information on patient level clinical characteristics that may confound the observed associations. The included studies provide minimal or no reporting on comorbidities (e.g., diabetes, cardiovascular disease, immunosuppression), behavioural factors (such as smoking), or baseline infection severity, all of which are known to influence the risk of hospitalisation, complications, and length of stay due to dental infections [42]. Similarly, details regarding surgical context such as difficulty and depth of impaction [43,44], operator experience [45], which have been reported to be implicated in complication rate following third molar surgery, are inconsistently reported or entirely absent. This limited clinical granularity introduces substantial residual confounding, making it difficult to disentangle whether hospitalisation is attributable to third molar status per se or to underlying patient risk profiles and management pathways.

In the included studies, the mean LOS from overall dental infections ranged from 2.4 days to 9 days [22,28]. These were calculated from the total number of dental hospitalisations, rather than 3M-specific data. Involvement of mandibular molars and M3M was reported to be correlated to an increase in LOS and ICU admission in only 3 studies [27,31,35]. Fu and colleagues reported the lower third molar to be implicated in 85.7% of ICU admissions in 2003–2004, and 64.4% of ICU admissions in 2013–2014, concluding that patients with M3M involvement were 6.5 times more likely to require ICU admission for care [31]. Jundt and colleagues found that M3M were implicated in 54.7% of hospitalisations and in 75% of cases where LOS was longer than 5 days, suggesting M3M involvement to be associated with more severe dental infections [35]. However, these were limited by the lack of adjustment for infection severity. Overall, differences in LOS between PPH arising from 3M and other teeth were not well reported (Table 3 and Table 4). One study reported that PPH for postoperative dental infection following prophylactic 3M removal was associated with a shorter mean LOS (6.7 days) compared to therapeutic extraction (7.9 days), or infections where the 3M was present on admission (7.1 days) [3]. One challenge while evaluating postoperative infections is the lack of published data on surgical difficulty, as the increasing surgical complexity and duration may increase the rate of complications [41]. A further point of debate in the management of M3M involves the potential role of coronectomy as a less invasive approach to complete extraction in select high risk cases. By removing the crown of the tooth and leaving the root undisturbed, this approach has been reported to reduce the incidence of iatrogenic nerve injury [46]. Complications have also been reported including root migration requiring intervention [47,48]. In the included studies in this review, root migration with secondary infection requiring hospitalisation were not reported.

Prophylactic removal of maxillary third molars remains a controversial topic in clinical practice, particularly as these teeth are often removed concurrently during the extraction of symptomatic or problematic mandibular third molars. Extraction of these teeth are associated with surgical trauma, postoperative swelling, pain, recovery time, and additional financial cost [49]. Conversely, retention of these teeth may also result in future pathology, such as the development of dentigerous cysts in unerupted teeth or periodontal compromise of the adjacent second molar due to overeruption of the maxillary third molar teeth [50]. Some authors have also reported removal of impacted third molars to be associated with a reduction in probing depths in both the maxilla and mandible [51,52]. In the present study, an interesting observation was the low complication rate associated with both the retention and removal of maxillary third molars. Although the available evidence remains limited, the potential benefits of intervention should be carefully discussed with patients and balanced against the potential surgical risks despite the currently limited data regarding their true incidence as well as the associated potential biological and financial cost when determining an appropriate patient-specific management strategy [14].

In this review, one mortality was reported by Kunkel et al. and Saito et al., two by Sahibzada et al., and five by Kityamuwesi et al. [26,34,38,40]. The implicated tooth was not described in these studies except for Kunkel et al., who reported a case of a 77-year-old with pre-existing coronary artery disease hospitalised for retromaxillary abscess from pericoronitis dying from myocardial infarction due to stent stenosis on a background of previous triple coronary artery disease 23 days following M3M removal under general anaesthetic [40].

The small sample size of most included studies was the main weakness of this research, with the overall number of included cases being disproportionately skewed to the publications by Rasteniene et al. and Wang et al. [25,26]. Limited data was also a major weakness of this study, with most studies describing data on dental infections in general, with limited description of variables relating specifically to the 3M teeth. In most studies, it is unclear whether admissions were due to postoperative complications or pre-existing conditions that necessitated hospital care, such as chronic pericoronitis, cysts, or systemic comorbidities. This confounding factor limits the ability to accurately attribute hospitalisations specifically to prophylactic or therapeutic 3M removal. The retrospective, audit-based nature of the included studies is another limitation. While clinical audits may reveal trends and patterns in treatment outcomes, unlike randomized clinical trials, they cannot infer causal relationships. The inclusion of only studies published in English also introduces potential language bias, as hospital-based odontogenic infection literature may be published in local or regional journals. A further important limitation is that while screening was undertaken by two reviewers, data extraction was not undertaken in duplicate, potentially introducing extraction error and bias. A major challenge relating to data collection for hospitalisations arising from 3M retention compared to perioperative complications of 3M removal is that complications from retained 3M arise over a longer timeframe and may not be captured in the data collection time frame of individual studies [3]. There was insufficient evidence to conclude whether the specific aetiology of dental caries, pericoronitis, postoperative infection or other causes of hospital admission arising from 3M, were correlated with increased patient morbidity and LOS. There was also insufficient evidence for age, gender, smoking status, comorbidities or time of presentation as predictors of patient outcomes when considering hospitalisations relating specifically to 3M teeth.

## 5. Conclusions

3M-related dental infections, including postoperative infections, appear to be a cause of oral health-related hospitalisation but remain inadequately described in the published literature. Only two studies reported third molar-specific hospitalisation with detailed aetiology and outcomes, and the remainder provided indirect attribution, with third molars accounting for 26.9% of hospitalisation within broader odontogenic infection cohorts. The M3M was reported to be more commonly involved than the maxillary 3M.

The overall level of evidence is low due to limitations in the existing literature, including the retrospective study designs and the small number of published cases specifically reporting hospitalizations related to third molars (3M). Most studies described hospitalizations from odontogenic causes more broadly, and individual studies reported data heterogeneously, with varying levels of detail regarding 3M. Among studies reporting potentially preventable hospitalizations (PPH), 26.9% involved 3M; however, definitions of PPH were not standardized across studies, and this figure should be interpreted as a descriptive proportion of hospitalizations in which third molars were implicated rather than as a population-level estimate of preventability. Insufficient information was available to assess the impact of demographic variables or to determine whether dental caries, pericoronitis, or postoperative infection following 3M removal were associated with increased length of stay.

The current evidence base is under-described, and future prospective studies with systematic data collection with sufficient case numbers to investigate specific demographic, aetiology, risk factors, comorbidities and other variables of PPH related to 3M are required.

## Figures and Tables

**Table 1:** Characteristics of included studies.

Year	Author	Country	Type of Study	Primary Outcomes Measures
2024	Ullah et al. [24]	Australia	Retrospective	Dental infection related hospital admission
2022	Wang et al. [25]	China	Retrospective	Dental infection related hospital admission
2022	Sahibzada et al. [26]	India	Retrospective	Dental infection related hospital admission
2022	Rasteniene et al. [27]	Lithuania	Retrospective	Dental infection related hospital admission
2019	Keswani et al. [28]	India	Retrospective	Dental infection related hospital admission
2019	Katoumas et al. [29]	Greece	Retrospective	Dental infection related hospital admission
2019	Nadig et al. [30]	UK	Retrospective	Dental infection related hospital admission
2018	Fu et al. [31] *	Australia	Retrospective	Dental infection related hospital admission
2015	Rasteniene et al. [32]	Lithuania	Retrospective	Dental infection related hospital admission
2015	Mucke et al. [33]	Germany	Retrospective	Dental infection related hospital admission
2015	Kityamuwesi et al. [34]	Uganda	Retrospective	Dental infection related hospital admission
2012	Jundt et al. [35]	USA	Retrospective	Dental infection related hospital admission
2012	Boffano et al. [36]	Italy	Retrospective	Dental infection related hospital admission
2012	Matthew et al. [37]	India	Retrospective	Dental infection related hospital admission
2011	Saito et al. [38]	Brazil	Retrospective	Dental infection related hospital admission
2011	Sanchez et al. [39]	Spain	Retrospective	Dental infection related hospital admission
2007	Kunkel et al. [40]	USA	Retrospective	3M related complications requiring hospital admission
2006	Flynn et al. [41]	USA	Retrospective	Dental infection related hospital admission
2006	Kunkel et al. [3]	USA	Retrospective	3M related complications requiring hospital admission

* Described 2 cohorts of dental infection related hospital admissions, 1 cohort from 2003–2004, and 1 cohort from 2013 to 2014.

**Table 2:** Studies with overlapping/duplicate cases.

Reference	Number of Cases	Number of Overlapping Cases	Retained Cases Count
Kunkel	100	54	Total number of unique cases—101 (third molar-specific data)
Kunkel	55		
Rastenienne	3509	285	Total number of unique cases = 3224 (broader odontogenic infection cohort)
Rastenienne	285		

**Table 3:** Study characteristics: third molar involvement, length of stay and aetiology data (odontogenic infection cohorts with partial attribution to third molars).

Year	Author	Mandibular 3rd Molar Involvement	Maxillary 3rd Molar Involvement	Other Teeth Involvement	Total Number of Patients	3rd Molars Related Mean LOS in Days (Range)	Any Tooth Related Mean LOS in Days (Range)	Aetiology—Pericoronitis of the 3rd Molar	Aetiology—Postoperative Infection After 3rd Molar Removal
2024	Ullah et al. [24]	12 (9.8%)	Not reported	90 (88.2%)	102	Not reported	2.7 ± 1.6	6 (5.9%)	Not reported
2022	Wang et al. [25]	85 (15.2%)	Not reported	664 (84.8%)	746	Not reported	9	113 (15.2%)	Not reported
2022	Sahibzada et al. [26]	13 (28.9%)	5 (14.3%)	82 (65%)	80	Not reported	8.5 (1–18)	Not reported	Not reported
2022	Rasteniene et al. [27]	975 (27.8%)	Not reported	2534 (72.2%)	3509	Not reported	7.9 ± 1.4 (1–44)	Not reported	Not reported
2019	Keswani et al. [28]	194 (56.2%)	Not reported	121 (43.8%)	315	Not reported	4 (3–10)	84 (26.7%)	Not reported
2019	Katoumas et al. [29]	37 (36.5%)	Not reported	65 (63.5%)	102	Not reported	5.9 ± 3.4 (2–27)	Not reported	7 (6.9%)
2019	Nadig et al. [30]	23 (15.9%)	0 (0%)	77 (84.1%)	100	Not reported	2.4 (0–13)	Not reported	Not reported
2018	Fu et al. [31] *	35 (67.3%)	Not reported	17 (32.7%)	52	Not reported	1.7 ± 0.5 (Year 2003–2004) 3.24 ± 4.1 (Year 2013–2014)	Not reported	Not reported
2015	Rasteniene et al. [32]	121 (42.2%)	Not reported	164 (57.8%)	285	Not reported	8.3 ± 4.9 (2–29)	Not reported	Not reported
2015	Mucke et al. [33]	59 (28.8%)	Not reported	146 (71.2%)	205	Not reported	7.1 ± 3.7	Not reported	Not reported
2015	Kityamuwesi et al. [34]	19 (29.7%)	Not reported	111 (70.3%)	130	3M involved in 75% of cases with LOS >5 days	Not reported	Not reported	Not reported
2012	Jundt et al. [35]	23 (54.7%)—not stated maxillary or mandibular 3M		19 (45.3%)	42	Not reported	4.57	Not reported	Not reported
2012	Boffano et al. [36]	33 (29.5%)	6 (5.4%)	73 (65.1%)	Not reported	Not reported	5.2 ± 3 (1–58)	Not reported	10 (8.9%)
2012	Matthew et al. [37]	49 (36%)	10 (7%)	78 (57%)	Not reported	Range 2–21	Not stated	Not reported	Not reported
2011	Saito et al. [38]	13 (14%)	1 (1.1%)	79 (84.9%)	Not reported	4.24 (1–31)	Range 2–21	Not reported	Not reported
2011	Sanchez et al. [39]	37 (26.6%)	Not reported	114 (73.4%)	7.3 (2–28)	7.3 (2–28)	4.24 (1–31)	9 (6%)	Not reported
2006	Flynn et al. [41]	25 (68%)	3 (8%)	9 (24%)	7.7 (2–16)	7.7 (2–16)	5.1 ± 3.0 (1–14)	8 (22%)	1 (3%)

* Described 2 cohorts of dental infection related hospital admissions, 1 cohort from 2003–2004, and 1 cohort from 2013 to 2014.

**Table 4:** Study characteristics: length of stay and aetiology data (third molar related admission cohorts).

Study	Mandibular 3rd Molar Involvement	Maxillary 3rd Molar Involvement	Other Teeth Involvement	Total Number of Patients	3rd Molars Related Mean LOS in Days (Range)	Aetiology—Pericoronitis of the 3rd Molar	Aetiology—Postoperative Infection After 3rd Molar Removal	Aetiology—Mandibular Fracture After 3rd Molar Removal	Aetiology—Intraoperative 3rd Molar Tooth Displacement into Anatomical Space	Aetiology—Postoperative Hemorrhage Following 3rd Molar Removal
Kunkel et al., 2007 [40]	100%—not stated maxillary or mandibular 3M		0 (0%)	100	7.3 (2–28)	29 (29%)	51 (51%)	11 (11%)	5 (5%)	1 (1%)
Kunkel et al., 2006 [3]	100%—not stated maxillary or mandibular 3M		0 (0%)	55	7.7 (2–16)	0 (0%)	45 (81.8%)	6 (10.9%)	1 (1.8%)	0 (0%)

## Data Availability

The raw data supporting the conclusions of this article will be made available by the authors on request.

## Supplementary Materials

The following supporting information can be downloaded at: https://www.mdpi.com/article/10.3390/dj14080503/s1, Table S1: PRISMA 2020 Checklist.
